# Importance of comprehensive genetic testing for patients with suspected vascular Ehlers–Danlos syndrome: a family case report and literature review

**DOI:** 10.3389/fgene.2023.1246712

**Published:** 2023-12-20

**Authors:** Xianda Wei, Xu Zhou, BoBo Xie, Meizhen Shi, Chunrong Gui, Bo Liu, Caiyan Li, Chi Zhang, Jiefeng Luo, Cundong Mi, Baoheng Gui

**Affiliations:** ^1^ Center for Medical Genetics and Genomics, The Second Affiliated Hospital of Guangxi Medical University, Nanning, Guangxi, China; ^2^ The Guangxi Health Commission Key Laboratory of Medical Genetics and Genomics, The Second Affiliated Hospital of Guangxi Medical University, Nanning, Guangxi, China; ^3^ The Second School of Medicine, Guangxi Medical University, Nanning, Guangxi, China; ^4^ Department of Rheumatology and Immunology, The Second Affiliated Hospital of Guangxi Medical University, Nanning, Guangxi, China; ^5^ Department of Ultrasound Diagnosis, The Second Affiliated Hospital of Guangxi Medical University, Nanning, Guangxi, China; ^6^ Department of Neurology, The Second Affiliated Hospital of Guangxi Medical University, Nanning, Guangxi, China

**Keywords:** vascular Ehlers–Danlos syndrome, vascular rupture, *COL3A1*, novel mutation, genotype–phenotype correlations

## Abstract

Vascular Ehlers–Danlos syndrome (vEDS), the most severe type of Ehlers–Danlos syndrome, is caused by an autosomal-dominant defect in the *COL3A1* gene. In this report, we describe the clinical history, specific phenotype, and genetic diagnosis of a man who died of vEDS. The precise diagnosis of this case using whole-exome sequencing provided solid evidence for the cause of death, demonstrating the practical value of genetic counseling and analysis. Early diagnosis for the proband’s son, who was also affected by vEDS, revealed initial complications of vEDS in early childhood, which have rarely been reported. We also reviewed the literature on *COL3A1* missense mutations and related phenotypes. We identified an association between digestion tract events and non-glycine missense variants, which disproves a previous hypothesis regarding the genotype–phenotype correlation of vEDS. Our results demonstrate the necessity of offering comprehensive genetic testing for every patient suspected of having vEDS.

## 1 Introduction

Ehlers–Danlos syndrome (EDS), also known as congenital connective tissue hypoplasia syndrome, is associated with defects in collagen synthesis and metabolism. EDS represents a class of collagen disorders among the wider group of heritable connective tissue diseases. Vascular EDS (vEDS) is a specific form of EDS caused by autosomal-dominant mutations in *COL3A1*. Although vEDS is generally associated with mild skin lesions, severe cardiovascular lesions can occur in rare cases. These lesions can progress to cause aortic dissection and aneurysm, which are prone to spontaneous rupture, leading to death. As such, vEDS is the most dangerous type of EDS and has the worst prognosis.

In this report, we describe the clinical history, unique phenotype, and genetic cause of a man who died from vEDS; his son was also affected by vEDS, as confirmed by whole-exome sequencing (WES). In addition, we reviewed the literature on *COL3A1* missense mutations and related phenotypes to provide more insight into the phenotype–genotype correlation in vEDS.

## 2 Clinical report

The proband was a 32-year-old man. In January 2021, he presented to a local hospital because of complaints of swelling of the left forearm and bulging of the blood vessels. He subsequently repeatedly visited doctors for systemic symptoms, including severe abdominal pain, lumbago, bulging vessels, sweating, a pale face, and transient amaurosis. Symptoms improved with a small dose of glucocorticoids and symptomatic treatment. Trauma caused by a violent impact was denied. In June 2021, he developed swelling of the right forearm; pain in the right forearm, back, and abdomen; and transient black spots in his vision. Ultrasound imaging revealed arterial abnormalities. Computed tomography (CT) angiography showed bilateral internal carotid and vertebral artery aneurysms, rupture of the left ulnar artery, and pseudoaneurysm.

**FIGURE 1:**
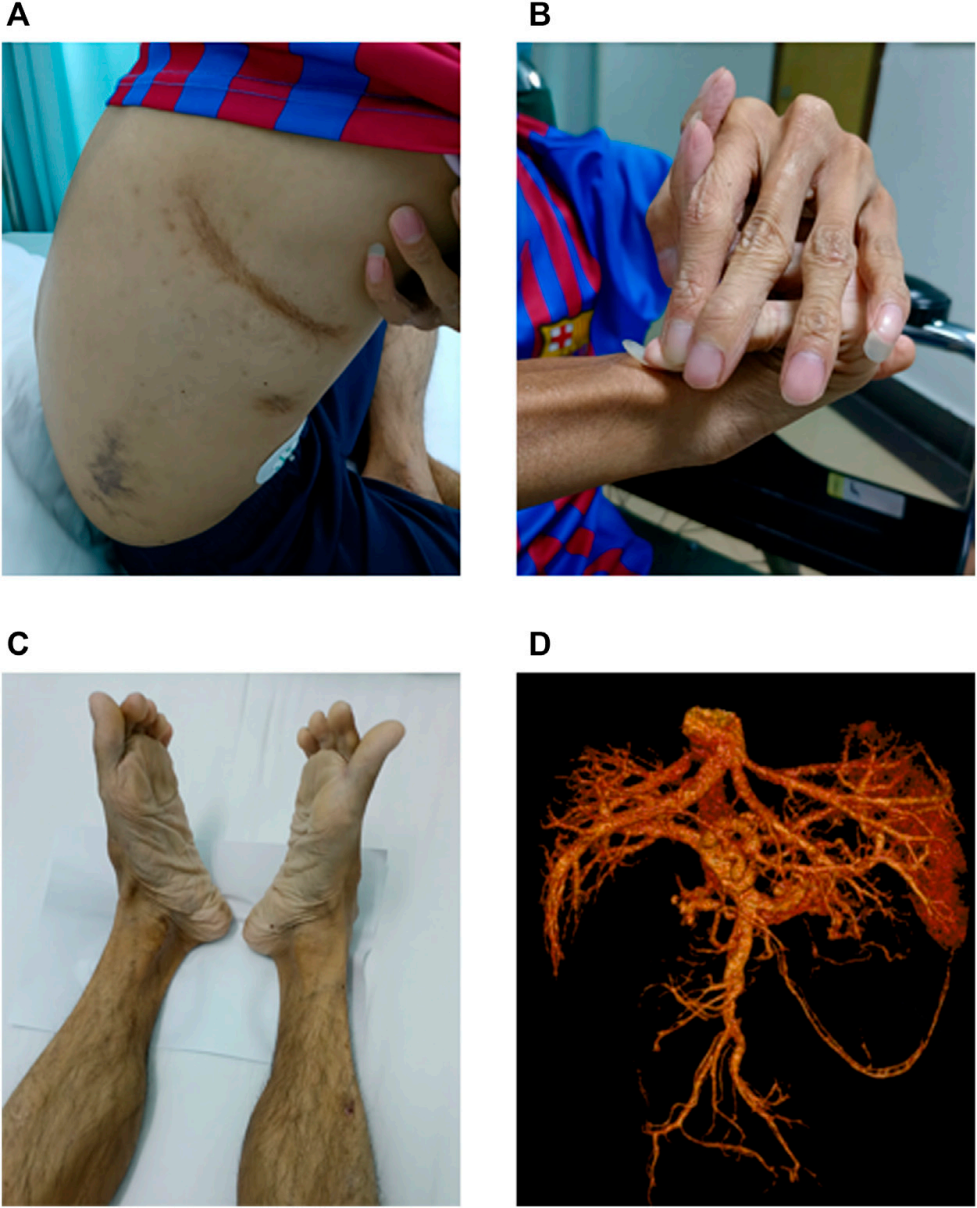
Main clinical findings of the proband. **(A)** Multiple ecchymoses (non-traumatic) in the anterior chest, right waist, and upper limbs. **(B)** Right finger with excessive dorsal flexion and the little finger pressed against the back of the hand. **(C)** Feet rotated inward. **(D)** Multiple aneurysms and aneurysmal dilation of the celiac trunk, proper hepatic artery, left hepatic artery, splenic artery, and both kidneys; arterial dissection of the celiac trunk and splenic artery is evident.

In July 2021, the patient visited our hospital with abdominal pain and black stools. Physical examination revealed a short height (150 cm) and low body weight (36 kg). He exhibited aging skin of the extremities, subcutaneous venous exposure, and multiple ecchymoses (non-traumatic) of the chest, right waist, and upper extremities (Figure 1A). His fingers were slender, and the joints were abnormally flexible. His right hand had excessive dorsal flexion and the little finger was pressed against the back of the hand (Figure 1B). His feet rotated inward (Figure 1C). Gastroscopy revealed no bleeding in the upper digestive tract. Vascular and abdominal CT (Figure 1D) revealed multiple aneurysms and aneurysmal dilation of the celiac trunk, proper hepatic artery, left hepatic artery, splenic artery, and both kidneys, along with arterial dissection of the celiac trunk and splenic artery. The patient experienced pain on percussion in the right renal area. Color ultrasound of the urinary system indicated mild hydrops in the right kidney, excluding the presence of urinary stones. Distal occlusion of the upper pole branch of the right renal artery, decreased perfusion of the right renal parenchyma, and a high density of fat sacs around the right kidney suggested bleeding and infarction of the right kidney. Kidney stones were further excluded by ultrasonography. Blood biochemistry tests, immunoglobulin (Ig)A/IgM/IgG, rheumatoid factors, autoantibody spectra, and cardiac color Doppler ultrasonography all exhibited negative findings. The patient died of arterial rupture and hemorrhagic shock 2 weeks after admission.

**FIGURE 2:**
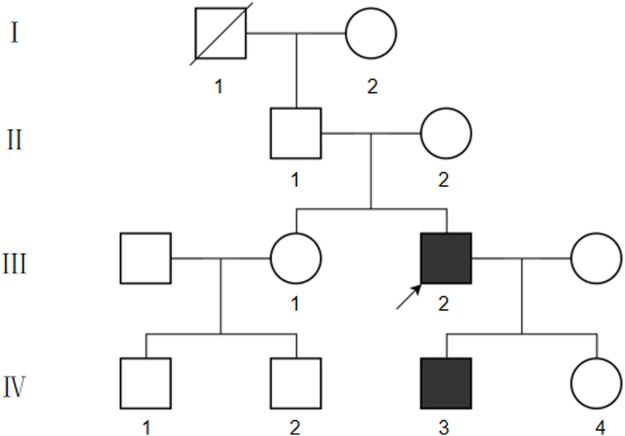
Pedigree chart of this case.

We collected basic information on the proband’s family members over three generations (Figure 2). The proband’s grandfather died of unknown cause, and the proband’s parents, sisters, and daughters did not show any abnormalities related to vEDS. However, the proband’s son, aged 3 years 4 months, had deep skin pigmentation, deep palm lines, poor wound healing of the skin, and occasional fresh blood in stools. Mosquito bites were also reported to be abnormally large at times. No surface hemangiomas were observed.

## 3 Genetic analysis

The rarity and complexity of the patient’s clinical manifestations implied the possibility of a genetic disease. To this end, we performed trio WES of the proband and his parents after genetic counseling; genetic testing was also performed on the proband’s son and proband’s older sister.

Genomic DNA was extracted from blood samples using a LabAid DNA kit (Zeesan Biotech Co., Ltd., Xiamen, China); target capture for WES (Human All Exon V5 Kit, Agilent Technologies, Foster City, CA, United States) and library sequencing (NextSeq CN500 platform, Illumina, San Diego, CA, United States) were conducted following the manufacturer protocols.

Data analysis and annotation were performed using the Genome Analysis Toolkit (GATK), version 3.4.0. The variants were identified and filtered using in-house protocols. The inclusion criteria for candidate variants were as follows: 1) heterozygous variants in an established causative gene of a dominant Mendelian disorder with a likely association to the patient’s phenotype; 2) variants with frequencies in the East Asian population in the gnomAD, NHLBI Exome Sequencing Project, 1000 Genomes Project, and in-house databases all <0.5%; and 3) computational evidence supports a deleterious effect of the variant. SpliceAI (Jaganathan et al., 2019), a deep-learning–based algorithm, was used to calculate the probability of splice-altering variants, providing a Δ score between 0 and 1. Candidate variants were verified by Sanger sequencing and true variants were validated following American College of Medical Genetics and Genomics/American Association of Molecular Pathology guidelines (Richards et al., 2015).

**FIGURE 3:**
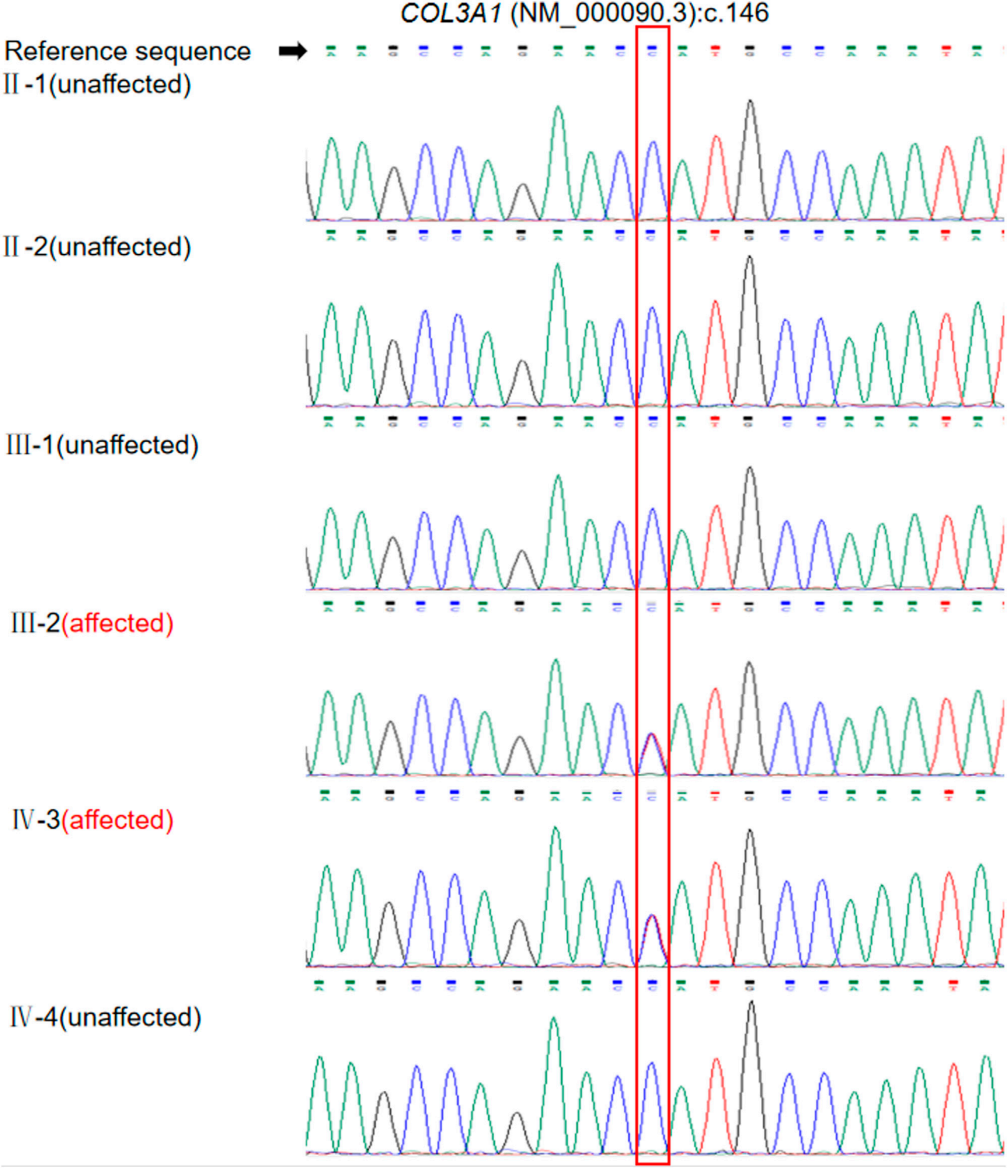
Validation of the *COL3A1* mutation c.146C>T in the patient and family members by Sanger sequencing. Annotation in the red box indicates the location of the mutation.

We identified a heterozygous missense variation, NM_000090.3:c.146C>T(p.Pro49Leu), in exon 2 of *COL3A1*, as the most likely genetic cause of the disease. The variant was heterozygous in the proband and his son and was absent in his parents and sister (Figure 3). The variant was classified as likely pathogenic according to the following criteria: 1) PS2: the variant was *de novo*, with both parental samples confirmed to be derived from the biological parents of the patient through single-nucleotide polymorphism analysis of trio-WES data; 2) PM2: this variant is not reported in the East Asian population database; and 3) PP2: the missense constraint Z-score of *COL3A1* in the Gnomad database was 4.09, indicating that missense variations in this gene are a common cause of disease. According to the calibration of computational tools for missense variant pathogenicity classification and ClinGen recommendations for PP3/BP4 criteria (Pejaver et al., 2022), the prediction results obtained with a single prediction software tool can be used as a basis for PP3 evidence. The single prediction tool utilized in our laboratory to determine the usage and strength of PP3 is revel. In this case, the Revel score for the *COL3A1* c.146C>T variant is only 0.586, which does not meet the criterion used for a PP3 level of supporting evidence (revel score ≥ 0.644). We also evaluated information about a previously reported variant c.145C>G p. (Pro49Ala) affecting the same residue as c.146C>T. Although homozygous c.145C>G variants have been detected in at least four patients with an autosomal recessive disorder caused by biallelic mutations in *COL3A1*, polymicrogyria with or without vascular-type EDS (OMIM 618343) (Vandervore, et al., 2017; Horn, et al., 2017), evidence supporting its pathogenicity in vEDS was insufficient. Thus, PM5 should not be applied to interpret c.146C>T(p.Pro49Leu) in our cases with vEDS.

Finally, we established a genetic diagnosis of vEDS for the patient and his son based on clinical and genetic findings.

## 4 Discussion

EDS is a heterogeneous group of connective tissue disorders that comprise a spectrum of monogenic conditions with multi-systematic and variable clinical manifestations (e.g., joint hypermotility, skin hyperextensibility, and tissue fragility) primarily affecting the skin, ligaments, joints, blood vessels, and internal organs. EDS is classified into 13 different subtypes, among which the vascular type (type IV) is the most severe and life-threatening, with arterial ruptures or dissections responsible for the majority of deaths. These events are unpredictable, and the fragility of the arterial walls often makes surgical repair difficult (Ruscitti et al., 2021).

The proband of this case had early symptoms, including peripheral vascular rupture events and skin abnormalities, which gradually evolved into thoracoabdominal vascular rupture. Although the clinical manifestations of the proband were in accordance with a diagnosis of vEDS, the rarity of this disease poses challenges for clinical diagnosis, especially for physicians in adult departments who may not have a strong awareness or sufficient knowledge of genetic disorders. We initially considered Marfan syndrome as a possible diagnosis, as the associated phenotypes overlap with those of vEDS, including aortic dissection, aortic aneurysm, and joint hypermotility. Following the advice of geneticists, we performed WES of the proband because of his multi-systematic and non-specific manifestations. The identification of a novel likely-pathogenic variation in *COL3A1*, c.146C>T, enabled a genetic diagnosis of vEDS for the patient. It should be noted that, though current evidences including the *de novo* occurrence and the absence in controls are sufficient to support the likely-pathogenicity according to the ACMG/AMP guidelines, functional evidences are still lacking. Further investigations of p.Pro49Leu mutant protein through *in vitro* or *in vivo* experiments will be vital to confirm the impact of this variant. In terms of this case, the diagnosis of vEDS with known prognosis eased the family’s concerns about the cause of death. Therefore, this case demonstrates the non-negligible practical value of genetic counseling and analysis.

Approximately two-thirds of published cases of genetically diagnosed vEDS are caused by point mutations in glycine residues (Ohyama et al., 2010). A single Gly substitution destabilizes the triple helix through a local disruption in hydrogen bonding and produces a discontinuity in the register of the helix (Brodsky and Persikov,2005).In *COL1A1*, *COL2A1* and *COL7A1*, Gly substitutions have been reported to cause more severe disorders than any other single-aminoacid mutations (Marini et al., 2007; Xu et al., 2020; Gupta et al., 2023).According to a review article, all gastrointestinal events originate from glycine substitutions, splicing variants, and in-frame indels, whereas variants that cause haploinsufficiency and non-glycine missense variants are not associated with gastrointestinal events (Frank et al., 2015). To gather the latest evidence and re-analyze the association between the *COL3A1* genotype and the vEDS phenotype, we conducted a literature review according to the following inclusion criteria: 1) reports of patients diagnosed with vEDS associated with only *COL3A1* mutation and 2) the mutation type is consistent with non-glycine missense mutations.

This search retrieved reports of 35 cases of vEDS. In contrast to the review article of Frank et al. (2015), three of our reviewed cases involved non-glycine missense variants, c.1351G>A p.(Glu451Lys), c.2791G>A p.(Glu931Lys), and c.3511G>A p. (Glu1171Lys), that were indeed associated with digestive tract symptoms (Table 1). In the present case, both the proband and his son experienced digestive tract symptoms. Thus, the present case and previous three non-glycine missense variants reported put into question the currently established relationships between the genotype of *COL3A1* and specific complications of vEDS. Accordingly, when performing genetic diagnosis for patients suspected as having vEDS, comprehensive testing of the *COL3A1* gene covering all types of variants (e.g., through next-generation sequencing) is preferred over targeted analysis of limited sites or regions (e.g., Sanger sequencing), regardless of the presence or absence of any specific symptoms.

**TABLE 1:** Associations of vascular Ehlers–Danlos syndrome (vEDS) mutation sites with clinical phenotypes.

Mutation	Age of initial onset* or age of diagnosis^#^ (years)	Thoracic and abdominal vascular events	Gastrointestinal events	Skin abnormalities	Distinct facial features^a^	Exercise and bone developmental abnormalities	Peripheral blood	Others	References
c.388G>T p.(Gly130Arg)	30^#^	+	−	−	+	+	−	+	Kroes et al. (2003)
c.721G>A p.(Glu241Lys)	0.75*	−	−	+	−	+	+	−	Ghali et al. (2019)
c.727G>A p.(Gly243Arg)	−	+	−	+	+	+	−	−	Gu et al. (2018)
c.781G>A p.(Gly261Ser)	−	+	−	−	−	−	−	+	Lee et al. (2008)
c.800G>T p.(Gly267Val)	−	+	−	−	+	−	+	+	Lipinski et al. (2009)
c.970G>A p.(Gly324Ser)	−	+	−	−	−	−	−	−	Rebelo et al. (2011)
c.1052G>T p.(Gly351Val)	−	−	+	−	−	+	+	+	Lumi et al. (2021)
c.1342G>A p.(Glu448Lys)	75	−	−	+	+	+	+	+	Colman et al. (2022)
c.1351G>A p.(Glu451Lys)	41*	−	+	+	−	−	−	+	Ghali et al. (2019)
c.1387G>A p.(Glu463Lys)	49	−	−	+	−	+	+	−	Colman et al. (2022)
c.1511G>A p.(Gly504Asp)	−	−	+	−	−	−	−	−	Nakagawa et al. (2015)
c.1862G>A p.(Gly621Glu)	−	+	−	−	−	−	+	+	Barboi et al. (2009)
c.1916G>T p.(Gly639Val)	−	+	+	−	−	−	+	−	Brightwell and Walker (2011)
c.1925G>A p.(Gly642Asp)	−	+	−	−	−	+	+	−	Ishiguro et al. (2009)
c.2044G>A p.(Glu682Lys)	UC	+	−	+	−	−	−	−	Ghali et al. (2019)
c.2194G>A p.(Gly732Arg)	9^#^	+	−	+	−	+	−	+	Kamalanathan et al. (2019)
c.2195G>T p.(Gly732Val)	−	+	−	+	−	−	−	−	Yang et al. (2007)
c.2321G>A p.(Gly774Asp)	−	+	−	−	−	−	−	+	De Sousa et al. (2020)
c.2465G>C p.(Gly822Ala)	−	−	+	−	−	−	+	−	Eder et al. (2013)
c.2512G>A p.(Gly838Ser)	4^#^	−	−	+	−	+	−	−	Narcisi et al. (1994)
c.2528G>A p.(Gly843Glu)	0.5*	+	−	+	−	+	−	+	Sadakata et al. (2010)
c.2644G>T p.(Gly882Cys)	−	+	−	−	−	−	−	+	Aydıner and Hançer (2020)
c.2791G>A p.(Glu931Lys)	18	−	−	+	−	−	−	−	Colman et al. (2022)
c.2791G>A p.(Glu931Lys)	16	−	+	+	−	+	−	−	Colman et al. (2022)
c.2896G>T p.(Gly966Cys)	−	+	−	+	+	+	−	−	Abrahamsen et al. (2015)
c.2932G>C p.(Gly978Arg)	−	+	−	+	−	+	−	−	Gu et al. (2018)
c.2959G>A p.(Gly987Ser)	−	−	−	+	+	+	−	+	Lan et al. (2018)
c.2996G>A p.(Gly999Asp)	37^#^	−	−	+	−	+	−	+	Gu et al. (2018)
c.3149G>T p.(Gly1050Val)	10*	−	−	−	−	+	−	+	Palmeri et al. (2003)
c.3175G>A p.(Gly1059Arg)	−	+	−	−	−	−	−	−	Sakai et al. (2019)
c.3440G>T p.(Gly1147Val)	UC^b^	−	−	+	−	+	−	+	Nuytinck et al. (1994)
c.3478A>G p.(Ile1160Val)	−	−	−	−	−	+	−	+	Ruscitti et al. (2021)
c.3511G>A p.(Glu1171Lys)	−	−	+	−	−	−	−	−	Ghali et al. (2019)
c.3554G>T p.(Gly1185Val)	−	−	+	−	−	−	−	+	Yang et al. (2022)
c.3851G>A p.(Gly1284Glu)	−	+	−	+	+	+	−	+	Jørgensen et al. (2015)

+, symptoms of the patient; −, patient does not have symptom or symptom is not reported; *, age of initial onset; #, age of diagnosis.

^a^ Includes thin vermilion of the lips, micrognathia, narrow nose, and prominent eyes.

^b^ UC, patient had symptoms in the neonatal period, but no specific age given.

The proband’s son, who carried the same *COL3A1* mutation, developed mild symptoms, including short stature, low body weight, skin pigmentation, deep palm prints, and poor skin healing. The boy also had black stool, indicating rupture of the blood vessels in the digestive tract. These observations suggest the initial complications of vEDS in early childhood but have rarely been reported in the published literature. In earlier reports, 25% of patients with vEDS had their first symptom by the age of 20 years and >80% had at least one symptom by the age of 40 years. Based on the high complication rates, the median age at the first major vascular event and at death for patients with vEDS are reported as 24.6 and 48 years, respectively (Pepin et al., 2000).

This early genetic diagnosis of the proband’s son (aged 3 years) presents an opportunity to assess the prognosis by referring to his father’s case, thereby enabling early interventions for management of the disease and prevention of further serious complications. For example, patients with vEDS require daily exercise and treatment. Care should be taken to avoid trauma (e.g., collision sports, heavy lifting, and extreme weight training). Arteriography should be discouraged and used only to identify life-threatening sources of bleeding before surgical intervention because of the risk of vascular injury (Byers, 1999).

In summary, this case highlights the importance of early and accurate diagnosis, genetic counseling, and avoiding high-risk activities and procedures in patients with vEDS (Frković et al., 2022). Thus, for late-onset genetic disorders such as vEDS, active utilization of genetic testing for younger relatives could create opportunities for early diagnosis of the disease, facilitating precise counseling and prevention of fatal disease progression. Moreover, the clinical findings and genetic analysis of this case could form the basis for the design of research programs, including further functional studies or modeling investigations.

## Data Availability

The datasets for this article are not publicly available due to concerns regarding participant/patient anonymity. Requests to access the datasets should be directed to the corresponding authors.
